# Development and Validation of a Site-Specific Tumor Burden Score for Predicting Surgical Outcomes in Advanced Ovarian Cancer

**DOI:** 10.3390/cancers17223649

**Published:** 2025-11-13

**Authors:** Zhiyang Xu, Xiaotian Li, Ying Liu, Yongqiang Tang, Weihuan Hou, Yihua Jin, Gaijing Cao, Lingxia Li, Hongxi Zhao, Xiaohui Lv, Shujuan Liu

**Affiliations:** 1 Department of Obstetrics and Gynecology, Xijing Hospital, Fourth Military Medical University, Xi’an 710032, China; xuzhiyy@163.com (Z.X.); lixt738602@163.com (X.L.); liuying1614@163.com (Y.L.); jinyihuaa@163.com (Y.J.); cgg77i8@163.com (G.C.); llxlemen@163.com (L.L.); 2 Department of Radiology, Xijing Hospital, Fourth Military Medical University, Xi’an 710032, China; tangyq12315@139.com (Y.T.); houweihuan@hotmail.com (W.H.); 3 Department of Obstetrics and Gynaecology, Tangdu Hospital, Fourth Military Medical University, Xi’an 710032, China; zhaohx@fmmu.edu.cn

**Keywords:** ovarian cancer, preoperative evaluation, tumor burden, suboptimal cytoreduction, predictive score

## Abstract

**Simple Summary:**

The goal of ovarian cancer (OC) preoperative evaluation is to assess surgical feasibility for achieving optimal cytoreduction. In this study, we systematically explored the role of site-specific tumor burden in the preoperative evaluation of OC and had several key findings: First, MRI was superior to CT for metastatic site-specific tumor burden assessment. Second, the tumor burden integrated suboptimal cytoreduction predictive score (AUC = 0.873) outperforms the metastatic site-integrated scores. Third, R0 resection was inversely linked to the predictive score and the tumor burden integrated predictive score <11 correlated with longer progression-free survival. We comprehensively delineate the role of site-specific tumor burdens in OC preoperative evaluation, which provides novel insights for refining preoperative assessment of ovarian cancer, marking a shift from evaluating metastatic sites to assessing metastatic site-specific tumor burden.

**Abstract:**

**Objective**: The relationship between surgical outcomes and metastatic sites in ovarian cancer (OC) is known, but the role of metastatic site-specific tumor burden remains unclear. **Methods**: We prospectively analyzed data from 202 OC patients. We developed a preoperative protocol evaluating tumor burden in 30 metastatic sites and created a predictive score for suboptimal cytoreduction, which was externally validated. **Results**: MRI-assessed tumor burdens demonstrated superior consistency with surgical findings compared to CT (κ = 0.4–1.0). Three site-specific tumor burdens (diaphragmatic spleen surface, hepatorenal recess, mesentery), upper abdominal tumor burden, and two clinical factors were identified as predictors of suboptimal cytoreduction. The predictive score incorporating these factors achieved an AUC of 0.873 (0.815 externally validated), outperforming metastatic site-integrated scores including the simulated Fagotti score (AUC: 0.656) and Suidan score (AUC: 0.8308). R0 resection rates were inversely correlated with predictive scores: 94.87% for scores of 0–3 versus 8.57% for scores >14. The peak of Youden’s index reached 11, and patients with predictive scores <11 had longer median progression-free survival. **Conclusions**: We demonstrated that site-specific tumor burden is correlated with surgical outcomes in OC. Incorporating tumor burden into preoperative assessment enhances prediction performance. We developed a clinically applicable tool, marking a shift from evaluating metastatic sites to assessing metastatic site-specific tumor burden.

## 1. Introduction

Ovarian cancer (OC) is the most lethal gynecologic malignancy [1]. Approximately 75% of OC patients are diagnosed at an advanced stage, with widespread peritoneal metastasis and a subsequent poor prognosis (5-year survival rate: 20–30%) [2]. The standard OC treatment is cytoreductive surgery combined with chemotherapy after surgery (primary debulking surgery, PDS) or before surgery (interval debulking surgery, IDS) [2]. The main goal of the surgical efforts is the removal of all macroscopically visible diseases which are associated with the best survival outcomes [3].

Patients who undergo PDS have improved survival compared with those who undergo IDS [4]. However, if an optimal or complete resection of the disease is not feasible, IDS is used to improve the chances of optimal resection without compromising overall survival [5]. Determining a suitable treatment for PDS or IDS on the basis of surgical ability, the perceived risk of complications, and the benefits of varying degrees of cytoreduction is crucial. Patients unsuitable for PDS should be identified, enabling them to benefit from IDS. Despite numerous attempts to use clinical and imaging data in evaluation models, these efforts have not yielded a validated tool that is widely used in clinical practice [6,7,8].

A key factor affecting surgical outcomes is the anatomic site of metastasis; however, few studies have focused on how site-specific tumor burden impacts surgical outcomes [9]. High tumor burden would increase surgical complexity and postoperative complications, thereby influencing surgical outcomes and patient prognosis in OC [10,11,12]. However, the precise preoperative assessment of tumor burden and its integration into preoperative evaluation models have not been sufficiently studied in OC.

Therefore, we conducted this retrospective analysis of prospectively collected data to investigate the role of integrating tumor burden into OC preoperative evaluation. Additionally, we developed standardized preoperative evaluation protocols that consider the tumor burden and compare the performance of different imaging modalities in evaluating the tumor burden in OC patients.

## 2. Methods

### 2.1. Study Design and Participants

For the discovery cohort, patients with suspected OC were prospectively enrolled from December 2018 to October 2023 in our hospital. The inclusion criteria were as follows: (1) aged 18–80 years; (2) had International Federation of Gynecology and Obstetrics (FIGO) stage III/IV disease; (3) had histopathologically confirmed epithelial ovarian carcinoma; (4) had a preoperative Magnetic Resonance Imaging (MRI) or Computed Tomography (CT) performed within 1 week before cytoreductive surgery; and (5) provided verbal informed consent. The exclusion criteria included concurrent malignancies, extra-abdominal metastases (e.g., brain, bone), poor physical condition (Eastern Cooperative Oncology Group, ECOG ≥ 3) [13], or incomplete data. We prospectively collected patients’ basic clinical information, preoperative imaging data, surgical records and followed up every 3 months after surgery in the first 2 years, and then every 6 months thereafter. An external validation cohort was retrospectively established from Tangdu hospital (November 2023–2024) using the same inclusion/exclusion criteria.

During the establishment of the patient cohort, some patients were considered unable to achieve complete resection before surgery. They were evaluated through multidisciplinary team discussions and determined to not benefit from PDS. In this study, all IDS patients received a standard paclitaxel-carboplatin regimen with a median of 3 NACT cycles (range: 2–3). Therefore, they received NACT followed by IDS. We collected imaging evaluations and clinical data before NACT and classified these patients into the non-R0 group only for the suboptimal cytoreduction prediction.

### 2.2. MRI and CT Acquisition and Evaluation

MRI scans were conducted using a 3.0T GE Discovery MR750 system (8-channel phased array coil) (GE Healthcare, Chicago, IL, USA), covering the diaphragmatic dome to the inferior pubic symphysis. CT imaging utilized a 64-slice LightSpeed VCT scanner (GE Healthcare, Chicago, IL, USA) with unenhanced and contrast-enhanced protocols. All imaging data were independently reviewed by two senior radiologists (20 and 10 years of experience). As all imaging reviews were performed preoperatively, the analysts were necessarily blinded to the final surgical outcomes, which were unknown at the time of assessment. Any discrepancies in interpretation were resolved by consensus between the two radiologists. (Supplementary Methods for detailed parameters).

### 2.3. Preoperative Imaging Evaluation Protocol

A preoperative imaging evaluation protocol was developed through multidisciplinary team evaluation, incorporating 30 anatomically defined metastatic sites (Table S1). These anatomical sites are not only common sites of metastasis in ovarian cancer but also potentially impact R0 resection. Lesion size was quantified via a 3-tier scoring system: 1 point (tumors < 0.5 cm), 2 points (0.5–5 cm), and 3 points (>5 cm or confluent disease) [14]. Our scoring system also considers the spatial distribution of the tumor burden. The tumor burden was stratified regionally: upper abdomen (diaphragmatic peritoneum, liver, stomach and spleen), middle abdomen (excluding lymph nodes), and lower abdomen (pelvic cavity and rectum). Regional scores were aggregated to reflect spatial tumor distribution. (Table S1). The predictive score for each patient was calculated as the total score of all metastatic sites except the primary site because it does not affect the outcome of the surgery. To compare the predictive performance of our scoring system with that of other scoring methods, patients were also evaluated according to the Suidan scoring system based on MRI examination results [15]. For the external validation cohort, the preoperative imaging protocols (MRI/CT) were deemed equivalent, meeting the same diagnostic requirements as our discovery cohort and providing sufficient resolution to assess all 30 anatomical sites. As all imaging assessments were based on reports generated preoperatively, analysts were blinded to the final surgical outcomes. Also we calculated PCI score using the preoperative imaging results [14].

### 2.4. Intraoperative Evaluation

Debulking surgeries (PDS/IDS) were performed by three gynecologic oncologists with >20 years of experience. Surgical procedures at Tangdu hospital were similarly performed by two senior gynecologic oncologists with over 15 years of experience. All cytoreductive surgeries (PDS and IDS) were performed via open surgery. While historical definitions of “optimal” cytoreduction (e.g., <1 cm residual disease) were used, the current standard of care and primary surgical goal is the complete removal of all visible diseases [3,15]. All intraoperative assessments align with the preoperative criteria. During the surgical procedure, the gynecologic oncologists performed a systematic intraoperative assessment, evaluating the same 30 anatomically defined metastatic sites and using the same 3-tier tumor burden scoring system that was used for the preoperative imaging evaluation (Supplementary Table S1). Surgical outcomes were classified into R0 (no macroscopic residual disease) or non-R0 (suboptimal cytoreduction) groups. IDS patients were assigned to the non-R0 cohort for predictive modeling and their intraoperative details were not included in the analysis. Patients were also evaluated according to the Fagotti laparoscopic scoring system based on surgical records to generate simulated Fagotti score [16]. The preoperative imaging protocols (MRI/CT) were deemed equivalent, meeting the same diagnostic requirements as our discovery cohort and providing sufficient resolution to assess all 30 anatomical sites.

### 2.5. Statistical Analysis

Statistical analyses utilized SPSS v27.0 (IBM). Continuous variables are reported as medians (minimum-maximum range) and categorical variables as frequencies (%). Integer-based scores are reported as median (interquartile range, Q1–Q3). Categorical data were compared using appropriate methods such as the χ^2^ test or Fisher’s exact tests. Continuous variables were assessed using Student’s *t*-test. Cohen’s weighted kappa analysis was performed to evaluate the consistency between the image score and the intraoperative findings. Receiver operating characteristic (ROC) curves were generated to determine the optimal cutoffs for age, Cancer Antigen 125 (CA-125), Human Epididymis Protein 4 (HE4), and Prognostic Nutritional Index (PNI) [17]. Ordinal univariate and multivariate logistic regression identified predictors of suboptimal cytoreduction in tumor burden factor and binomial univariate and multivariate logistic regression were used for clinical factors. Variables with a *p*-value < 0.05 in the univariate analysis were considered candidates for multivariate analysis. To avoid high multicollinearity, a forward selection logistic regression analysis was performed separately for individual site factors (combined with clinical factors) and for regional/total scores. To create a clinically applicable scoring system, the ORs of the final predictors were rounded to the nearest simple integers to serve as weights. The model’s calibration was assessed using the Hosmer-Lemeshow goodness-of-fit test. ROC curve analysis and the area under the curve (AUC) were used as indicators of the models’ predictive accuracy. Progression-free survival (PFS) was analyzed using the Kaplan–Meier method, and survival curves were compared between two groups by the log rank test.

## 3. Results

### 3.1. Patient Characteristics

**Figure 1:**
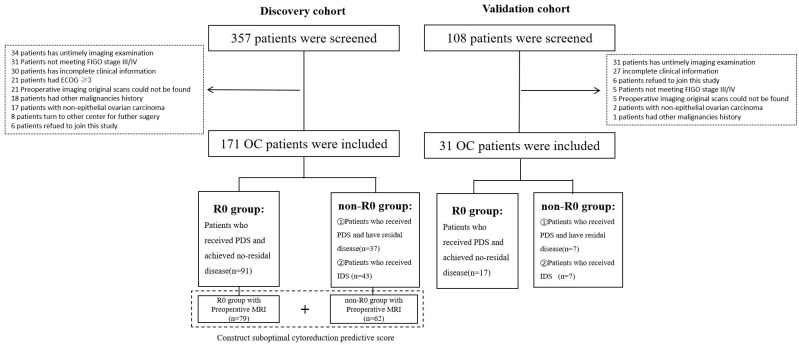
Study overview.

The discovery cohort included 171 OC patients from Xijing hospital, and the external validation cohort included 31 OC patients from an external hospital (Figure 1). In the discovery cohort, 128 patients received PDS, and 71.09% (91/128) achieved R0 resection. The remaining PDS patients (28.91%, 37/128) and all IDS patients (*n* = 43) were grouped into the non-R0 group (Table 1). In the validation cohort, 17 and 14 patients were grouped into the R0 and non-R0 groups, respectively (Table S2).

We used a standardized preoperative evaluation protocol to measure all the patients’ tumor burdens in the discovery cohort based on preoperative imaging data and obtained MRI score and CT score, respectively (Table S1). The median preoperative MRI scores were 10.11 ± 7.64 and 17.91 ± 9.09 for patients in the R0 group and non-R0 group, respectively, with a statistically significant difference (*p* < 0.001). The median preoperative CT scores were 11.84 ± 6.73 and 16.53 ± 11.43 in the R0 group and non-R0 group, respectively, with a statistically significant difference (*p* < 0.001).

### 3.2. Comparisons Between Preoperative MRI/CT Site-Specific Tumor Burdens and Intraoperative Observations

**Figure 2:**
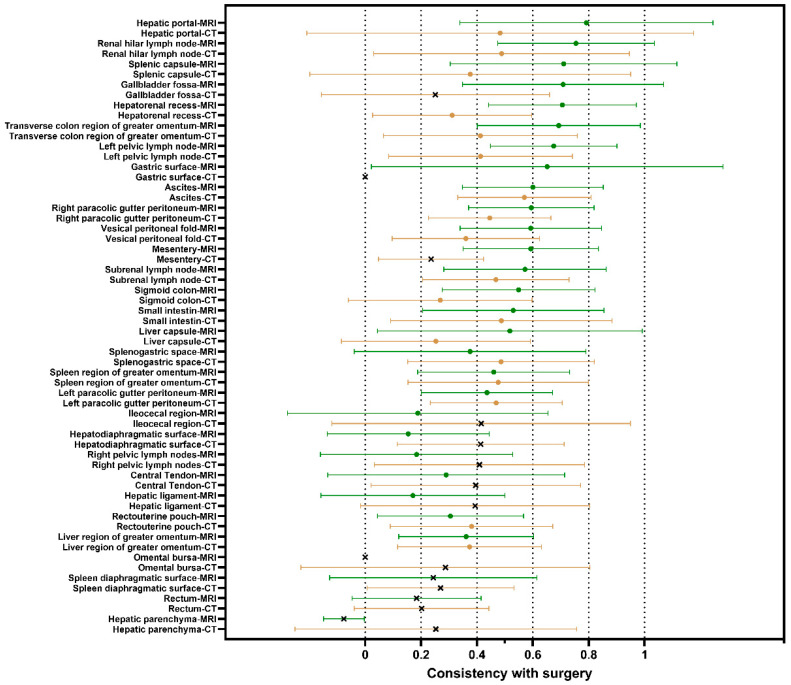
Consistency between preoperative MRI/CT tumor burden and intraoperative observations at different metastatic sites. The green line represents the MRI results and the brown line represents the CT results. Circular markers indicate statistical significance (*p* < 0.05), while “×” markers indicate no statistical significance (*p* > 0.05). Markers represent Cohen’s weighted kappa value, and the horizontal whiskers represent the 95% confidence. Low-consistency ranges: 0.2–0.4; Medium consistency ranges: 0.4–0.6; High-consistency ranges: 0.6–1.

An accurate preoperative assessment is therefore an important prerequisite for selecting the best treatment strategy, especially for site-specific tumor burden. We evaluated the consistency between the preoperative tumor burden and intraoperative observations in patients who underwent both preoperative MRI and CT examinations. Compared with intraoperative observations, 18 site-specific tumor burdens had consistency with statistical significance (*p* < 0.05). Compared to CT, MRI exhibited a better consistency with surgical findings in 76.2% of site-specific tumor burdens (13/18), particularly within the medium-to-high consistency (κ = 0.4–1.0; 13/16 sites). In the low-consistency ranges, except for three areas (splenogastric space, spleen region of greater omentum and the left paracolic gutter peritoneum), MRI also showed higher consistency (Figure 2).

Furthermore, we repeated this analysis in a larger sample size. MRI demonstrated moderate consistency (κ = 0.4–0.8) with intraoperative assessments at 73.33% of the sites (22/30), whereas CT showed lower consistency (κ = 0.2–0.4) at 70.0% of sites (21/30). Both CT and MRI exhibited weak consistency in deep intestine-related regions, such as mesentery and ileocecal region. Among the three areas where CT assessments were initially superior to MRI in the paired patients, only splenogastric space maintained higher CT consistency than MRI after expanding the sample size (Figures S1 and S2). These results indicate that MRI has greater accuracy in preoperative site-specific tumor burden assessment in OC patients.

### 3.3. Determination of the Suboptimal Cytoreduction Related Site-Specific Tumor Burden

To determine the role of site-specific tumor burden in suboptimal cytoreduction prediction, univariate and multivariate analyses of 30 different MRI site-specific tumor burdens, tumor burden spatial distributions and clinical factors were performed. Univariate analysis revealed that three clinical parameters (CA-125 ≥ 560 U/mL, HE4 ≥ 150 pmol/L and PNI ≤ 45.7) and 11 MRI site-specific tumor burdens were significantly associated with suboptimal cytoreduction (*p* < 0.05). The tumor burden on the diaphragmatic surface of spleen and mesentery were the most predictive criteria for suboptimal cytoreduction. Specifically, for a 1-point increase in the diaphragmatic surface of the spleen tumor burden score, the risk of suboptimal cytoreduction increases by 7.243 times (OR 7.243, 95% CI 3.111–16.866). For the spatial distribution of tumor burden, the upper abdomen had an OR of 1.278 (95% CI 1.149–1.421) (Table 2).

After forward selection in multivariate analysis, 2 clinical and 4 site-specific tumor burdens remained significantly associated with incomplete cytoreduction (Table 2). The clinical criteria were a CA-125 level ≥ 560 U/mL (OR 2.925, 95% CI 1.147–7.646) and a PNI ≤ 45.7 (OR 2.898, 95% CI 1.090–7.707), while the radiological criteria were site-specific tumor burden in the diaphragmatic surface of the spleen (OR 3.970, 95% CI 1.528–9.962), hepatorenal recess (OR 2.053, 95% CI 1.140–3.696), mesentery (OR 2.468, 95% CI 1.132–5.380) and upper abdominal total score (OR 1.278, 95% CI 1.149–1.421) (Table 2).

### 3.4. Evaluating the Performance of the Tumor Burden-Integrated Suboptimal Cytoreduction Predictive Score

**Figure 3:**
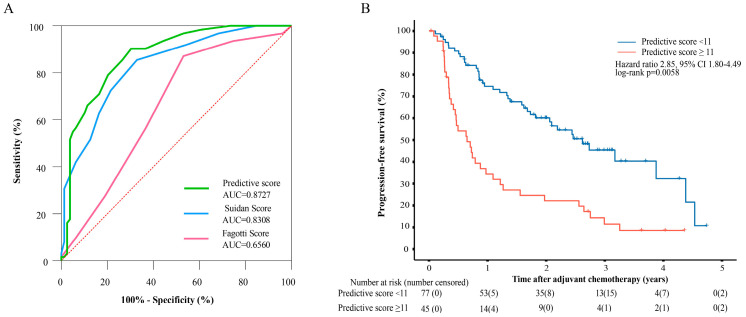
The performance of the tumor burden-integrated predictive score (**A**) ROC curves were generated to evaluate the performance of different models in predicting surgical outcomes. (**B**) Progression-free survival of patients grouped by tumor burden-integrated predictive score.

Moreover, we developed a tumor burden-integrated suboptimal cytoreduction predictive scoring system and validated it in an external cohort. Each of the 6 significant multivariate logistic regression factors was assigned a specific weight derived from their ORs (Table S3). In the discovery cohort, the median predictive score was 8 (0–44), and the AUC for R0 resection was 0.8727 (Figure 3A). In the external validation cohort, the model yielded an AUC of 0.815 (95% CI: 0.635–0.931) (Figure S3). The predictive scores showed an inverse relationship with R0 resection rates: 94.87% (score 0–3), 70.27% (4–8), 43.33% (9–14), and 8.57% (>14) (Table S4). The peak of the Youden’s index reached 11, suggesting that this cutoff may best balance misclassification risks for treatment decision-making. Longitudinal follow-up data revealed that patients who exceeded this critical threshold (score ≥ 11) demonstrated significantly inferior progression-free survival compared to those who were below the cutoff (HR = 1.98, 95% CI: 1.21–3.25; log-rank *p* < 0.0058; Figure 3B). Furthermore, to ensure a fair comparison with the original CT-based Suidan score, we performed a sub-analysis on all patients who underwent preoperative CT (*n* = 67). In this cohort, the CT-based Suidan score yielded an AUC of 0.734 which also supports the superior performance of our MRI-based SSTB model.

We further assessed model calibration and clinical utility. The model demonstrated good calibration in both cohorts. In the discovery cohort, this was confirmed by a non-significant Hosmer-Lemeshow test (χ^2^ = 11.082, *p* = 0.197) and a low Brier score of 0.1467. This good calibration was maintained in the validation cohort, which also showed a non-significant Hosmer-Lemeshow test (χ^2^ = 12.507, *p* = 0.085) and a low Brier score of 0.1795. Furthermore, decision-curve analysis (DCA) demonstrated that our predictive score provided a superior net benefit across a wide range of clinically practical threshold probabilities in both the discovery (approx. 15–85%) and validation (approx. 15–82%) cohorts indicating clinical applicability (Supplementary Figure S4).

We compared the predictive performance of our tumor burden-integrated model and other models that considered only tumor sites (Fagotti score, Suidan score) for suboptimal cytoreduction. The median simulated Fagotti score was 6 (IQR: 3–8) and 8 (IQR: 6–10) for patients in the R0 group and non-R0 group, respectively, with statistically significant differences (*p* < 0.001). The median Suidan score was 3 (IQR: 1–4) and 7 (IQR: 4–9) for patients in the R0 group and non-R0 group, respectively, with statistically significant differences (*p* < 0.001). Our tumor burden-integrated predictive score demonstrated superior performance (AUC = 0.8727) compared with the simulated Fagotti score (AUC = 0.6560) and the Suidan score (AUC = 0.8308). Furthermore, to ensure a fair comparison with the original CT-based Suidan score, we performed a sub-analysis on all patients who underwent preoperative CT (*n* = 67). In this cohort, the CT-based Suidan score yielded an AUC of 0.734 which also supports the superior performance of our model. Furthermore, our tumor burden-integrated predictive score also demonstrated superior predictive accuracy for suboptimal cytoreduction compared to the retrospectively calculated Peritoneal Cancer Index (AUC = 0.7435) (Figure S5).

## 4. Discussion

In this study, we investigated the role of site-specific tumor burden in OC preoperative evaluation. Our findings demonstrated that MRI had better accuracy in evaluating site-specific tumor burden. Three site-specific tumor burdens and upper abdomen tumor burdens emerged as predictors of suboptimal cytoreduction. We developed a system integrating site-specific tumor burden parameters, which demonstrated superior predictive performance for suboptimal cytoreduction compared with metastatic site-involved models. These findings provide novel insights and facilitate a new quantifiable decision-making tool for OC precision therapy.

Accurate noninvasive detection remains the cornerstone of preoperative evaluation. While the 2010 ESUR guidelines recommended CT for OC staging, emerging modalities such as MRI and radionuclide imaging have demonstrated potential value in preoperative assessments [18,19,20]. In this study, we compared CT and MRI, the two most widely available imaging modalities, for evaluating the tumor burden. Analysis of paired preoperative scans and intraoperative observations demonstrated the superior consistency of MRI findings with intraoperative findings, particularly in anatomically complex regions such as the mesentery. While both modalities aligned with surgical observations, MRI showed higher overall consistency rates. Notably, MRI maintained consistency advantages over CT even in challenging deep-seated areas. These findings suggest that MRI provides enhanced precision for evaluating tumor burden in complex anatomical regions during OC preoperative assessment.

The goal of OC preoperative evaluation is to identify features predictive of suboptimal cytoreduction. Initially, focused on specific metastatic sites, the prediction of suboptimal cytoreduction later expanded to include clinical factors linked to suboptimal outcomes [15,16]. Tumor burden, a key feature of metastasis, is correlated with surgical resectability in different cancers [10]. For example, the Peritoneal Cancer Index, which quantifies abdominal tumor burden spatially, is applicable to resectability assessment and prognostic prediction in gastric, colorectal, and ovarian cancers [9,21]. In this study, we integrated the tumor burden with the metastatic site to represent site-specific tumor burden. Among the 30 site-specific tumor burdens, the splenic diaphragmatic surface, hepatorenal recess, mesentery, and upper abdomen tumor burdens were associated with suboptimal cytoreduction. The mesenteric and upper abdominal burdens reflected both the technical complexity and increased tumor load, which is consistent with prior location-focused studies [22]. Splenic diaphragmatic burden, unlike that in hepatic regions, strongly indicated systemic disease severity [23], while the hepatorenal recess remains surgically critical because of its deep anatomic location and risk of recurrence [24]

Currently, there is still a lack of effective preoperative assessment tools for OC. Although many predictive indices have been developed, their performance often shows limited generalizability across medical teams [19,25]. This variability may arise from differences in institutional expertise and evolving surgical competencies over time, which can alter the criteria and contributing factors for suboptimal cytoreduction. We systematically assessed the tumor burden across 30 OC-specific metastatic sites, which significantly surpassed the Suidan score (18 sites) and other existing scores (typically around 20 sites) [15,26]. And we compared the difference in our predictive score and other scoring systems in Table S5. This granular anatomical mapping not only enhances surgeons’ preoperative understanding of disease distribution but also enables tailored surgical planning based on individual skill levels and institutional resources. Furthermore, the protocol enables the development of center-specific simplified scoring systems that can be iteratively updated with advancing surgical expertise. This study provides an accessible framework for less-experienced surgeons to standardize preoperative assessments while allowing dynamic refinement of predictive models to align with evolving surgical proficiency. These features address critical gaps in current OC preoperative evaluation paradigms, particularly in resource-diverse clinical settings.

A key strength of our study is its direct clinical applicability and adaptability. We propose an accessible 30-site assessment framework that enhances, rather than replaces, existing ESGO guidelines. While ESGO recommends comprehensive preoperative imaging to guide the critical PDS versus IDS triage decision, it lacks a standardized, quantitative protocol for its interpretation. Our SSTB protocol helps fill this gap, providing a non-invasive tool (AUC 0.873) that outperformed existing scores. Notably, the performance of our straightforward, clinically interpretable score is comparable to that of recent, highly complex radiomics models [27,28]. Furthermore, we acknowledge that any specific predictive cutoff (11 in our cohort) may vary between institutions, reflecting differences in institutional expertise and evolving surgical competencies. The primary utility is therefore the framework itself, which enables other centers to standardize their preoperative evaluations and, critically, to develop and iteratively update their own center-specific predictive models to align with their advancing surgical proficiency.

There are several limitations to our study. This was a retrospective analysis of prospectively collected data and, thus, was subject to some biases, particularly selection bias. A further limitation is the retrospective calculation of the Peritoneal Cancer Index (PCI). As PCI was not part of our original prospective protocol, its retrospective assessment was solely for post hoc comparison and may be subject to inherent documentation biases compared to a prospectively planned PCI calculation during surgery. Although all procedures were performed by highly experienced gynecologic oncologists at both centers, potential surgeon-related variability in surgical decision-making and aggressiveness cannot be fully excluded. As both the discovery and external validation cohorts were from a single country, the generalizability of our specific predictive score to other healthcare systems or ethnic populations may be limited. While our validation cohort’s imaging was assessed by experienced local radiologists, our study lacked a centralized radiologic review where the same radiologists assessed images from both cohorts, which could introduce inter-observer variability. Patients were chosen for surgery based on traditional clinical decision-making and we included the features before neoadjuvant chemotherapy for IDS patients. We considered these features which represent the true positive features of suboptimal cytoreduction and included them only in predictive score development. In addition, our current study did not incorporate quantitative radiomics features. A promising future direction would be to create a hybrid model that integrates our intuitive, site-specific tumor burden score with agnostic deep learning or radiomics features extracted from the same MRI scans [29]. A further limitation is that our study could not address the important relationship between initial site-specific tumor burden and the optimal number of NACT cycles. This remains a critical and understudied topic. Future studies are warranted to investigate whether preoperative disease burden, perhaps quantified by our site-specific tumor burden score, might help guide the optimal duration of NACT to improve patient selection for IDS.

## 5. Conclusions

We systematically explored the role of site-specific tumor burden in the preoperative evaluation of OC and demonstrated that site-specific tumor burden correlates with surgical outcomes in OC patients. Compared with traditional metastatic site-based models, the predictive system that integrates site-specific tumor burden exhibited superior performance. We also propose clinically applicable tools that consider site-specific tumor burden for OC preoperative assessment.

## Figures and Tables

**Table 1:** Patients Characteristics.

	R0 Group	Non-R0 Group	*p* Value
Number of patients	91 (53.22%)	80 (46.78%)	-
Age (years)	57 (20–79)	56 (33–76)	0.108
BMI (kg/m^2^)	23.27 (17.85–34.24)	22.66 (14.06–31.98)	0.112
CA125 (kU/L)	379.15 (24.15–7055)	852 (19.25–11619)	0.887
HE4 (pmol/L)	286 (42.8–1500)	248.7 (42.7–1393)	0.059
VEGF (pg/mL)	395.47 (24.48–1291.29)	516.41 (48.32–1311.41)	0.048
PNI	46.2 (28.85–58.6)	45.9 (28.5–56)	0.284
Pathologic subtypes			0.539 *
High-grade serous carcinoma	79 (86.82%)	70 (87.50%)	-
Low-grade serous carcinoma	2 (2.20%)	4 (5.00%)	-
Mucinous carcinoma	4 (4.39%)	2 (2.50%)	-
Endometrioid carcinoma	4 (4.39%)	1 (1.25%)	-
Clear cell carcinoma	2 (2.20%)	3 (3.75%)	-
FIGO stage			0.170
III	73 (80.22%)	57 (71.25%)	-
IV	18 (19.78%)	23 (28.75%)	-
Imaging examination type before treatment			0.091
MRI only	59 (64.84%)	39 (48.75%)	-
CT only	12 (13.19%)	18 (22.50%)	-
MRI and CT	20 (21.98%)	23 (28.75%)	-

Continuous variables are represented by the median (minimum-maximum range). * Pathologic subtypes other than high-grade serous carcinoma were combined for chi-square test due to small sample sizes.

**Table 2:** Multivariate model of significant clinical and MRI criteria predictive of residual disease.

	Univariate Analysis			Multivariate Analyze			Predictive Score
	OR	95% CI	*p*	OR	95% CI	*p*	
Age ≥ 55.5	0.68	0.348–1.327	0.258				
CA125 ≥ 560	4.044	1.995–8.201	0.001	2.925	1.147–7.464	0.025	2
HE4 ≥ 150	2.726	1.164–6.385	0.021				
PNI ≤ 45.7	9.000	4.091–19.801	0.001	2.898	1.090–7.707	0.033	2
Diaphragmatic surface of liver	1.651	1.142–2.388	0.008				
Diaphragmatic surface of spleen	7.243	3.111–16.866	0.001	3.970	1.582–9.962	0.003	3
Central tendon of diaphragm	1.987	1.149–3.436	0.014				
Falciform ligament of liver	1.901	1.111–3.255	0.019				
Gallbladder fossa	3.136	1.644–5.979	0.001				
Hepatorenal recess	2.596	1.614–4.176	0.009	2.053	1.140–3.696	0.01	2
Splenic capsule	2.661	1.520–4.657	0.001				
Splenic hilum	2.264	1.473–3.481	0.001				
Omentum	1.550	1.063–2.261	0.023				
Mesentery	4.797	2.501–9.198	0.001	2.468	1.132–5.380	0.023	2
Small intestine	1.639	1.007–2.668	0.047				
MRI total score	1.103	1.052–3.157	0.001				
Upper abdominal	1.278	1.149–1.421	0.001	1.278	1.149–1.421	0.001 *	1
Middle abdominal	1.273	1.111–1.458	0.001				
Lower abdominal	1.100	0.970–1.247	0.138				

* To mitigate the impact of multicollinearity between variables, a multivariable analysis was performed on the combined MRI total score, Upper abdominal score, Middle abdominal score, and Lower abdominal score. Only one parameter showed statistical significance in the multivariate analysis.

## Data Availability

The original contributions presented in this study are included in the article and Supplementary Materials. Further inquiries can be directed to the corresponding authors.

## Supplementary Materials

The following supporting information can be downloaded at: https://www.mdpi.com/article/10.3390/cancers17223649/s1, Figure S1: Consistency between preoperative MRI findings and intraoperative observations in different metastatic sites; Figure S2: Consistency between preoperative CT findings and intraoperative observations in different metastatic sites; Figure S3: ROC curve analysis for predictive score in validation cohort; Figure S4: Decision-Curve analysis of Discovery cohort and validation cohort; Figure S5: ROC curve analysis for Peritoneal Cancer Index in discovery cohort; Table S1: Ovarian cancer preoperative evaluation system; Table S2: Patients Characteristics in validation cohort; Table S3: Ovarian cancer suboptimal cytoreduction prediction score; Table S4: Outcomes of debulking surgery based on the predictive model; Table S5: Comparison of Predictive Scoring Systems for Suboptimal Cytoreduction.
