# Supplementary material for: Development and Validation of a Site-Specific Tumor Burden Score for Predicting Surgical Outcomes in Advanced Ovarian Cancer
Source: Cancers (Basel). 2025 Nov 13;17(22):3649. doi: 10.3390/cancers17223649 (PMC12651799; doi:10.3390/cancers17223649)
Supplement: Supplementary file 1 [file cancers-17-03649-s001.zip › cancers-3938159-supplementary.pdf]

Development and Validation of a Site-Specific Tumor Burden Score for Predicting Surgical Outcomes in Advanced Ovarian Cancer

Contents

Supplementary methods 2

Supplementary Figure S1. Consistency between preoperative MRI findings and intraoperative observations in different metastatic sites 4

Supplementary Figure S2. Consistency between preoperative CT findings and intraoperative observations in different metastatic sites 5

Supplementary Figure S3. ROC curve analysis for predictive score in validation cohort 6

Supplementary Figure S4. Decision-Curve analysis of Discovery cohort and validation cohort. 7

Supplementary Figure S5. ROC curve analysis for Peritoneal Cancer Index in discovery cohort 8

Supplementary Table S1 Ovarian cancer preoperative evaluation system 9

Supplementary Table S2 Patients Characteristics in validation cohort 11

Supplementary Table S3 Ovarian cancer suboptimal cytoreduction prediction score 12

Supplementary Table S4 Outcomes of debulking surgery based on the predictive model 13

Supplementary Table S5 Comparison of Predictive Scoring Systems for Suboptimal Cytoreduction 14

Supplementary methods

MRI and CT acquisition and evaluation

MR examinations were performed on GE Discovery MR750 3.0T scanner with an 8-channel phased array coil. Patient fasted for 6-8 hours and underwent bowel preparation before examinations. Scanning range was from the level of the diaphragm to the lower edge of the pubic symphysis.

The MR protocol for the abdomen consisted of the axial-in-phase T1-weighted sequence (TR 200ms, TE 2.37ms, Slice sickness 6mm, gap 1.2mm, Voxel size 1.2×1.2×6.0mm, NEX 1); axial fat-suppression T2-weighted sequence (TR 2200ms, TE 86ms, Slice sickness 6mm, gap 1.2mm, Voxel size 1.1×1.1×6.0mm, NEX 1); coronal T2-weighted sequence (TR 1400ms, TE 91ms, Slice sickness 6mm, gap 1.2mm, Voxel size 1.6×1.6×6.0mm, NEX 1); enhanced axial T1-weighted sequence (TR 4.49ms, TE 2.19ms, Slice sickness 3mm, gap 0.6mm, Voxel size 1.2×1.2×3.0mm, NEX 1); enhanced coronal T1-weighted sequence (TR 3.80ms, TE 1.27ms, Slice sickness 3mm, gap 0.6mm, Voxel size 0.6×0.6×3.0mm, NEX 1); enhanced sagittal T1-weighted sequence (TR 3.80ms, TE 1.27ms, Slice sickness 3mm, gap 0.6mm, Voxel size 0.6×0.6×3.0mm, NEX 1); and diffusion-weighted imaging sequence (TR 4300ms, TE 57ms, Slice sickness 6mm, gap 1.2mm, Voxel size 1.2×1.2×6.0mm, NEX 7, b=1000 s/mm2).

The MR protocol for the pelvis consisted of axial T1-weighted sequence (TR 2630ms, TE 70ms, Slice sickness 5mm, gap 1mm, Voxel size 0.4×0.4×5.0mm, NEX 2); axial fat-suppression T2-weighted sequence (TR 5060ms, TE 62ms, Slice sickness 5mm, gap 0.6mm, Voxel size 0.6×0.6×3.0mm, NEX 2); coronal fat-suppression T2-weighted sequence (TR 4800ms, TE 70ms, Slice sickness 4.5mm, gap 1mm, Voxel size 0.9×0.9×5.0mm, NEX 2); sagittal fat-suppression T2-weighted sequence (TR 4800ms, TE 70ms, Slice sickness 4.5mm, gap 0.9mm, Voxel size

0.4×0.4×4.5mm, NEX 2); enhanced axial T1-weighted sequence (TR 650ms, TE 8.3ms, Slice sickness 5mm, gap 1.0mm, Voxel size 0.7×0.7×5.0mm, NEX 1); enhanced coronal T1-weighted sequence (TR 650ms, TE 8.3ms, Slice sickness 4.5mm, gap 0.9mm, Voxel size 0.7×0.7×4.5mm, NEX 1); enhanced sagittal T1-weighted sequence (TR 650ms, TE 8.3ms, Slice sickness 4.5mm, gap 0.9mm, Voxel size 0.7×0.7×4.5mm, NEX 1); and diffusion-weighted imaging sequence (TR 3900ms, TE 65ms, Slice sickness 5mm, gap 1.0mm, Voxel size 1.4×1.4×5.0mm, NEX 7, b=800s/mm2).

CT examinations were all performed on multi-slice CT scanners. All patients were scanned preoperatively with a 64-slice spiral computed tomography (CT) system (Light-Speed VCT, GE Healthcare, American) for unenhanced and contrast-enhanced scanning. Acquisition parameters were as follows: 100 kVp, maximum tube current 350 mA, collimation 0.625 mm, rotation speed 0.5 s, and a pitch of 1. Automatic exposure control and current modulation was used with this equipment. The images were reconstructed with a standard soft-tissue kernel. The contrast-enhanced CT examinations were composed of hepatic arterial phase, hepatic parenchymal phase and delayed phase. Using the contrast agent tracking technique, the 5s in the aortic plane was delayed to select the ROI to monitor the CT value, and when the CT value reached 100HU, the delayed 5s automatically triggered the scan of the hepatic arterial phase; in the hepatic parenchymal phase, images were acquired 30 seconds after the hepatic arterial phase; in the delayed phase, images were acquired 3-5 minutes after administration of contrast media.The contrast agent was iodide 370mgI/ml, injection rate of 3ml/s, dose 0.7ml/kg.

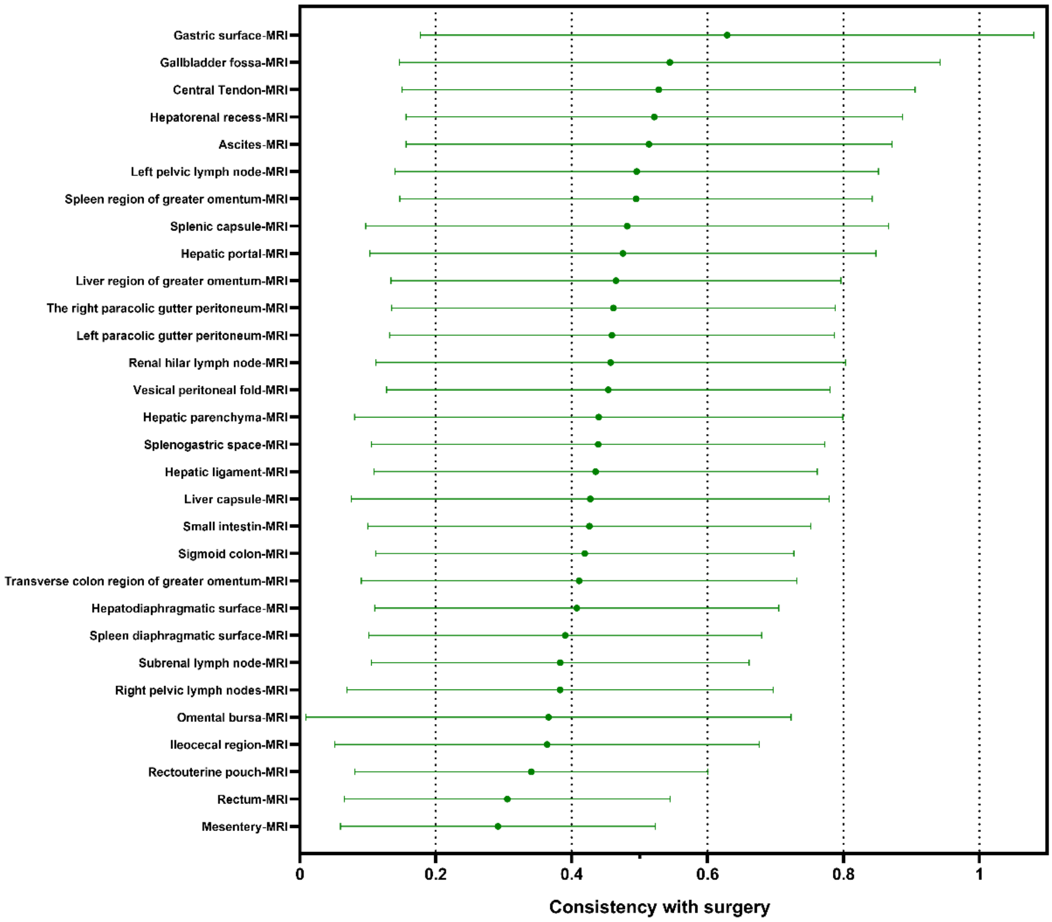

Supplementary Figure S1. Consistency between preoperative MRI findings and intraoperative observations in different metastatic sites. Lesion size scores of 30 metastatic sites assessed by preoperative MRI ranked in descending order of consistency with intraoperative observations(n=106). Circular markers indicate statistical significance (P<0.05)

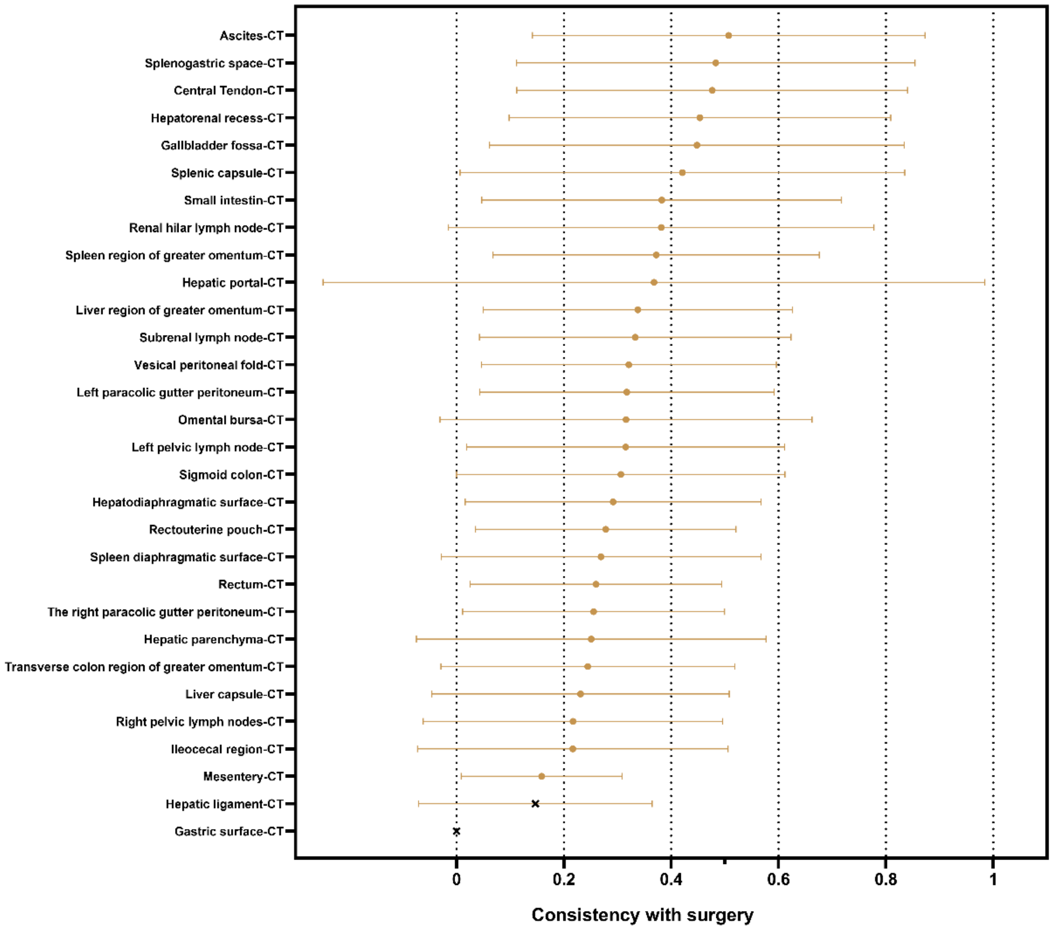

Supplementary Figure S2. Consistency between preoperative CT findings and intraoperative observations in different metastatic sites. Lesion size scores of 30 metastatic sites assessed by preoperative MRI ranked in descending order of consistency with intraoperative observations(n=53). Circular markers indicate statistical significance (P<0.05), while "×" markers indicate lack of statistical significance (P>0.05).

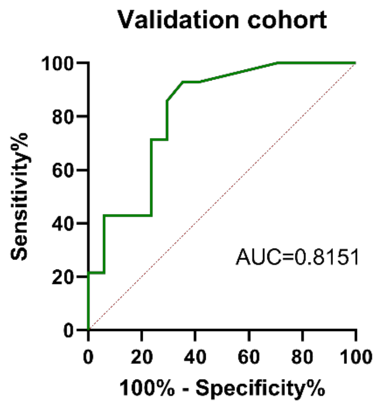

Supplementary Figure S3. ROC curve analysis for predictive score in validation cohort. ROC curves were generated to evaluate the performance of predictive score in validation cohort. The corresponding AUC values for each model are listed in the graph.

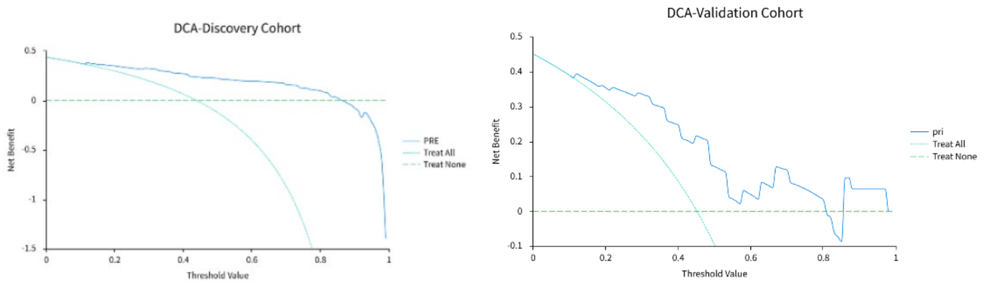

Supplementary Figure S4. Decision-Curve analysis of Discovery cohort and validation cohort.

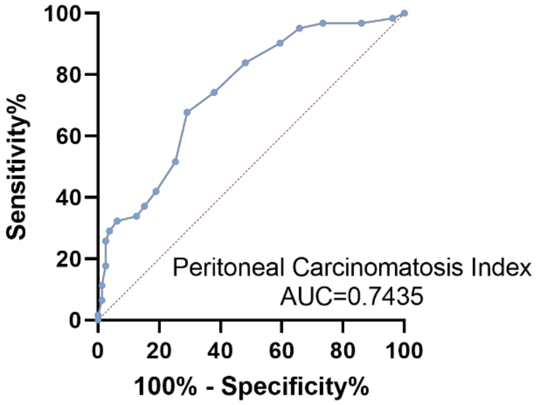

Supplementary Figure S5. ROC curve analysis for Peritoneal Cancer Index in discovery cohort.

Supplementary Table S1 Ovarian cancer preoperative evaluation system

Location	Lesions	Preoperative			Interoperative			
		Size (cm)	Lesion Score[*]	Description	Size (cm)	Lesion Score[*]	Description	Residual disease
Diaphragmatic peritoneum	Hepato diaphragmatic surface							
	Spleen diaphragmatic surface							
	Central Tendon							
Liver	Liver capsule							
	Hepatic portal							
	Hepatic ligament							
	Gallbladder fossa							
	Hepatorenal recess							
	Hepatic parenchyma							
Stomach	Gastric surface							
	Omental bursa							
Spleen	Splenic capsule							
	Splenogastric space							
Upper abdominal total Lesion size Score[#]								
Greater omentum	Liver region							
	Spleen region							
	Transverse colon region							
Intestine	Mesenteriolum							
	Small intestine							
	Ileocecal region							
	Sigmoid colon							
Abdominal Peritoneum	Paracolic gutter peritoneum(left)							
	Paracolic gutter peritoneum (right)							
Middle abdominal total Lesion size Score[#]								
Pelvic cavity	Rectum							
	Rectouterine pouch							
	Vesical peritoneal fold							
Lower abdominal total Lesion size Score#								
Adnexal region	Left							
	Right							
Lymph nodes	Renal hilar lymph node*							
	Subrenal lymph node							
	Pelvic lymph node (left/reght)							
Ascites	Moderate / large amount							

* **Lesion Score calculation:** The Lesion Score was calculated by the tumour diameters. 1 point: 0-0.5 cm; 2 point: 0.5-5 cm; 3 point: ≥ 5cm or confluent disease

\# **The three different spital total lesion score calculation:** The spital total lesion score can be calculated easily by adding the above Lesion Score.For detail: the Upper abdominal contains Diaphragmatic peritoneum, Liver, Stomach and Spleen. Lower abdominal contains Pelvic cavity and Rectum. The rest was divided into middle except Lymph nodes.

Supplementary Table S2. Patients Characteristics in validation cohort

	R0 group	Non-R0 group
**Number of patients**	17	14
**Age (years)**	58(42-73)	55(45-71)
**CA125 (kU/L)**	288(12.1-1352)	569(42.4-1161)
**HE4 (pmol/L)**	152(513-1500)	271.5(50.52-1500)
**PNI**	47.2(28-59)	46.95(31-53.15)
**Pathologic subtypes**		
High-grade serous carcinoma	17	14
**FIGO stage**		
III	14	10
IV	3	4

The median is shown in the table.

Supplementary Table S3. Ovarian cancer suboptimal cytoreduction predictive score

**Parameters**	**Predictive Score**
CA125 ≥ 560	2
PNI ≤ 45.7	2
Spleen diaphragmatic surface	Lesion size Score (0-3) ×3
Hepatorenal recess	Lesion size Score (0-3) ×2
Mesentery	Lesion size Score (0-3) ×2
Upper abdominal	Upper abdominal total Lesion size Score
**Total**	**Sum of all parameter scores**

Supplementary Table S4. Outcomes of debulking surgery based on the multivariate predictive model

Score	Total patients	R0 resection	Non-R0 resection
0-3	39(27.66%)	37(94.87%)	2(5.13%)
4-8	37(26.24%)	26(70.27%)	11(29.73%)
9-14	30(21.28%)	13(43.33%)	17(56.67%)
> 14	35(24.82%)	3(8.57%)	32(91.43%)

Supplementary Table S5. Comparison of Predictive Scoring Systems for Suboptimal Cytoreduction

Feature	Site-Specific Tumor Burden Score	Suidan Score	Fagotti Score
**Assessment Modality**	Preoperative Imaging (MRI/CT)	Preoperative Imaging (MRI/CT)	Diagnostic Laparoscopy
**Core Principle**	Quantifies site-specific Tumor Burden (Lesion Size)	Identifies Site Involvement (Yes/No)	Identifies Site Involvement (Yes/No)
**Scoring System**	lesion size score (1-3) per site	Site-based predictive score	Site-based predictive score
**Number of Sites Evaluated**	Systematically assesses **30** anatomical site	Assesses **8** anatomical sites	Assesses 7 key laparoscopic sites
